# The Dual Effect of Transformational Leadership on Individual- and Team-Level Performance: The Mediational Roles of Motivational Processes

**DOI:** 10.3389/fpsyg.2021.606066

**Published:** 2021-03-17

**Authors:** Hairong Lu, Feng Li

**Affiliations:** ^1^CAS Key Laboratory of Behavioral Science, Institute of Psychology, Chinese Academy of Sciences, Beijing, China; ^2^Department of Psychology, University of Chinese Academy of Sciences, Beijing, China; ^3^Department of Psychology, Education, and Child Studies, Erasmus University Rotterdam, Netherlands

**Keywords:** dual-level transformational leadership, team efficacy, team process, self-efficacy, multilevel

## Abstract

Using matched four-stage data from 477 team members and their 132 team leaders in Chinese companies, we examined a cross-level model in which group- and individual-focused transformational leadership (TFL) and their influence on team and member performance from the perspective of multilevel model of motivation in teams. The results indicated that group-focused TFL exerts positive effects through sequential mediation of team efficacy and team process whereas individual-focused TFL has a positive effect on team members' performance through sequential mediation of followers' self-efficacy and individual regulation process. In addition, we also find significant cross-level mediation effects demonstrating that group-focused TFL was positively related to self-efficacy through the mediator of team efficacy, team efficacy was positively related to the individual regulation process through the mediator of the team process, team process was positively related to individual performance through the mediator of the individual regulation process. Theoretical and applied implications are discussed.

## Introduction

Work teams have been used more and more broadly in modern companies, as people assume that working with a team can be more productive than work individually. However, process loss during teamwork may undermine the effectiveness of the teams due to the failure in achieving individual and team goals. Researchers argued that the problems of process loss largely result from lacking motivation (Kerr and Bruun, 1983). In organizational settings, leaders are likely to play a key role in motivating followers and promoting team performance (Zaccaro et al., 2008). As we know, leadership and team motivation are both multilevel phenomena (Gardner et al., 2020). Therefore, a multilevel model is needed to understand motivational effects of leadership in teams.

By distinguishing behaviors addressing individual differences and the team as a whole, dual-level transformational leadership (TFL) was developed to provide a new perspective to understand leadership effectiveness in teams (Wang and Howell, 2012; Kark and Shamir, 2013). It refers to transformational leadership focused on individual and team levels simultaneously. Individual-focused TFL is characterized by fully considering the individual differences between followers, providing specific coaching, and attaching expectations accordingly. Group-focused TFL aims to exploit the potential of the team and develop shared values and beliefs of the team goal. Studies try to explore the effects of dual-level transformational leadership from different perspectives. For example, leader/team-member exchange (Chun et al., 2016), knowledge sharing (Dong et al., 2017), person–environment fit (Klaic et al., 2018), and trust in different levels (Braun et al., 2013), are demonstrated to play roles in the effects of dual-level TFL. However, understandings with regard to this concept are far more from comprehensive, due to the limited understanding of the detailed motivational process of it.

As one of the most important functions of leadership is to motivate people, and lack of motivation may cause process loss and ineffectiveness of teams (Kerr and Bruun, 1983), studies examining the motivational effects of dual-level TFL are needed. However, after reviewing the literature, we find that discussions of the detailed motivational mechanism of dual effects of TFL are quite preliminary (Akinlade, 2014; Pourbarkhordari et al., 2016; Bormann and Diebig, 2020). Wang and Howell (2012) and Tung (2019) demonstrated that group-focused and individual-focused TFL could influence team and individual performance through arousing team member's group identification and leader identification, respectively. Windlinger et al. (2020) and Lau (2014) found that dual-focused TFL were positively related to collective efficacy and individual efficacy, respectively. However, they failed to take into account the motivational regulation process, which is deemed as an inseparable part of the motivation in teams. Therefore, further exploration of the motivational effects of dual-level TFL will advance theories of leadership and teamwork.

Drawing from Chen and Kanfer's (2006) multilevel model of motivation in teams, we assume that the motivation effects of TFL can be both horizontal (effects unfolding with episodes) and vertical (effects functioning cross-level). In particular, we argue that individual-focused TFL behaviors conveying some information about personal recognition can promote team members' self-efficacy. Individuals with high self-efficacy tend to set a high goal for themselves and put more effort to complete it and, thus, experience a productive individual regulation process. A productive individual regulation process could no doubt result in high individual performance. At the team level, group-focused TFL behavior emphasizing team value and making an effort to build team member's group identity could boost team efficacy. Team members with high team efficacy mean that they trust their coworkers and believe in their capability; thus, the team will experience a high-quality team process and result in a high level of team performance. Moreover, we propose cross-level relationships in the current model to explore the cross-level effects of team-level structures in predicting individual performance.

In testing the proposed model, this study contributes to the extant literature in several ways. First, based on the dual-level TFL model and Chen and Kanfer's (2006) cross-level model of motivation in teams, the present study could extend the line of multilevel research to the domain of transformational leadership by exploring the influence processes of TFL at both individual and team levels simultaneously and examining cross-level relationships between the team level and the individual level. We noticed that Wu et al. (2010) tested the dual-level TFL model by examining the positive influencecs of group-focused TFL and negative influences of difference in individual-focused TFL in teams, both at the team level. However, their focus is only on the team level. In contrast, our current research directly examines the multilevel and cross-level effects of TFL. Besides, the TFL behaviors in our study included two additional dimensions: team-building behavior at the team level and communicating high expectations at the individual level. With a wider variety of TFL behaviors, our research could provide a more comprehensive picture of the influences of dual-level TFL on followers (Wang and Howell, 2010). More recently, Wang and Howell (2012) also proposed and demonstrated a similar parallel model of dual-level TFL, but they focused on the effectiveness of dual-level TFL through employee identification at different levels, and efficacy was only one of the outcome variables. Furthermore, none of these two studies included the individual regulation process or team process, although these behaviors are important components in the team motivational process.

Second, to our knowledge, this is the first study to test Chen and Kanfer's (2006) full model of motivation in teams. We can find in previous studies that only part of the model proposed in Chen and Kanfer (2006) was tested. For example, Chen et al. (2007) and Cai et al. (2019) found that motivational states like employee felt empowerment and self/team-efficacy play roles in the team motivation model. Similarly, Curcuruto et al. (2020) found a positive relationship between leadership related antecedent factors and the goal regulation process in a safety field. However, none of them tested both the motivational states and the subsequent regulated process as a whole as specified in the original model proposed by Chen and Kanfer (2006). The present study contributes beyond that literature by including both the motivational states and goal regulation process specified in the multilevel model of motivation in teams and testing a full model of the dual motivational effect of TFL.

## Theories and Hypothesis

### Dual-Level Transformational Leadership

In organizations, leaders are expected to motivate individual followers and enhance team performance (Chen et al., 2007; Yammarino and Dansereau, 2008). Multilevel studies showed that indicators of individual and team performance are quite different (Chen, 2005; Braun et al., 2013). Following this trend, the concept of dual-level TFL, which distinguishes individual-focused TFL behavior and group-focused TFL behavior, was created to distinguish leader–follower interaction and leader–team interaction (Wang and Howell, 2010; Kark and Shamir, 2013). Individual-focused TFL is characterized by fully considering the individual differences between followers, providing specific coaching, and attaching expectations accordingly. Group-focused TFL aims to exploit the potential of the team and develop shared values and beliefs of the team goal (Wang and Howell, 2010; To et al., 2015). It is characterized by leaders who pay equal attention to and treat identically all group members. There are four dimensions in the individual-focused TFL: (a) communicating high expectations, (b) follower development, (c) intellectual stimulation, and (d) personal recognition. The dimensions of team-focused TFL include (a) emphasizing group identity, (b) communicating group vision, and (c) group-building.

Empirical evidence from Wang and Howell (2010, 2012) study supported the structure of this dual-level model and showed that individual-focused TFL is positively related to leader identification, personal initiative, and task performance, while team-focused TFL is positively related to team identification, helping behaviors, and team performance. In addition, evidence from top manager teams, R&D teams, and some mixed teams support the effectiveness of dual-level TFL in predicting team effectiveness and individual performance, respectively, and jointly (Chun et al., 2016; Dong et al., 2017; Lorinkova and Perry, 2018). Overall, this dual-level model of TFL overcomes the shortcomings of traditional transformational leadership and provides a new perspective for understanding leadership effectiveness in a multilevel way.

### Multilevel Model of Motivation in Teams

Work motivation is depicted as a multifaced, intrapsychic process that affects employee's work attitudes, behaviors, and work performance (Kanfer, 1990). Based on social cognitive theory and open system theory, Chen and Kanfer (2006) developed a multilevel model of motivation in teams to explain the motivational process in and of teams. The main point of this model is that there are functional similarities in the motivational process at individual and team levels, and those motivational concepts are proposed to have cross-level effects.

In this model, they first identified several parallel constructs of the motivation process and relationships at both the individual and team levels. That is, motivational states influence individual and team processes, such as what an individual (or team) chooses to do (i.e., goal generation, team transition process) and how an individual (or team) tries to accomplish that goal (i.e., goal striving, team action process) (Marks et al., 2001). Second, they proposed cross-level effects between individual- and team-level motivation. Third, a series of antecedents and outcomes are considered in the model. For example, the leadership and organizational climate are antecedents of motivational states, and performance is an outcome of the motivational process (Chen et al., 2009, 2011). We propose that this model is well-suited to explain the complex motivational mechanism of dual-level TFL.

### Individual-Level Effects of Transformational Leadership

A large number of studies suggested that individual-level TFL could enhance employee performance (Braun et al., 2013). A meta-analysis has shown that the overall validity of individual-level TFL is ~0.44 (Judge and Piccolo, 2004). Some researchers argue that the relationship between leadership and employee performance is not direct, and motivational factors are important mediators through which leadership take effects (Chen et al., 2011). Thus, we argue that individually focused TFL influence individual performance through a motivational process.

Self-efficacy is defined as the belief in ones' capabilities to organize and execute the courses of action required for a specific task, and it is a core concept of individual-level motivational states. It is argued in the multilevel model of motivation in teams that contextual factors like leadership will influence employee's self-efficacy. According to social cognitive theory, an individual's self-efficacy could be built by verbal persuasion (Bandura, 1977). Thus, it can be expected that individual-focused transformational leaders could enhance followers' self-efficacy by communicating high expectations and expressing personal recognition (Lau, 2014). Other individual-focused TFL behaviors, such as follower development and intellectual stimulation, can also help build followers' confidence in their abilities to accomplish certain tasks by developing their skills and knowledge. The positive relationship between TFL and followers' self-efficacy has gained lots of support (Chen and Bliese, 2002; Aggarwal and Krishnan, 2013). For example, it was demonstrated empirically by Windlinger et al. (2020) that individual-focused TFL positively predicts teacher's efficacy in a school setting.

According to Chen and Kanfer's (2006) motivation model, individuals with high efficacy are motivated to have a better goal generation and goal-striving process and finally yield better individual performance. This line of reasoning can also refer to social cognitive theory, which states that individuals with high self-efficacy tend to set more challengeable goals, formulate strategy, and accordingly put forth more effort to accomplish that goal (Locke and Latham, 2002; Gollwitzer and Oettingen, 2011). Theories and literature suggest that goal generation and goal-striving process are usually closely interrelated (Chen and Kanfer, 2006), such that goal generation sets the stage and affects the initiation, direction, intensity, and persistence of goal striving. Conversely, obstacles in goal striving may influence goal commitment and subsequent goal generation (Locke and Latham, 1990). Referring to Chen et al. (2005), we combine goal generation with goal striving and name it as an individual regulation process. It indicates how individuals act and react in the pursuit of goals. Evidence from Chen et al. (2005) gave support for the mediating role of the individual regulation process between self-efficacy and individual performance. Overall, we suggest a two-step hypothesis at the individual level:

**Hypothesis 1**. Self-efficacy and the individual regulation process sequentially mediate the positive influence of individual-focused TFL on individual performance.

### Team-Level Effects of Transformational Leadership

As depicted in the dual-level TFL model, group-focused transformational leaders motivate the team as a whole through behaviors such as emphasizing group identity, communicating a group vision, and team building. Those behaviors intend to create a coordinating and cooperating atmosphere in teams and facilitate team member's identification with their team. Studies of team leadership have shown that TFL is positively associated with team performance (Wang and Howell, 2010; Dong et al., 2017).

Further, in accordance with the effects of individual-focused TFL, we proposed a parallel functional pathway of group-focused TFL effects. Group-focused TFL exerts its effects on team performance through a series of team motivational processes. Similar to individual-level effects, team-level motivational states such as team efficacy play key roles in this process. Team efficacy is defined as team members' shared sense of the team's ability to organize and execute a specific task. Group-focused TFL behaviors such as team building, group vision communication, and highlighting group identity could enhance team members' confidence in their teams in accomplishing a specific task. A group of studies suggested that transformational leadership is positively related to team efficacy (Schaubroeck et al., 2007; Walumbwa et al., 2008; Wu et al., 2010). In addition, evidence from a longitudinal study indicates that TFL behavior could promote team potency (Sivasubramaniam et al., 2002).

According to the multilevel model of motivation in teams, team efficacy has a positive effect on team performance through the team goal generation and team goal-striving process (corresponding to individual-level construct goal generation and goal striving) and finally yielding a result of better team performance (Marks et al., 2001; Chen and Kanfer, 2006). Teams with a high level of team efficacy are likely to set more challenging team goals and accordingly put forth more effort to accomplish them. Team efficacy can influence the way team members set their team goals, what strategies and plans they choose, how to allocate resources, and how much effort they exert. Eventually, it influences team performance (Chen et al., 2005; Grant, 2015). Meta-analyses provide strong support for the relationship between team efficacy and team performance, yielding a correlation of 0.41 (Gully et al., 2002) and 0.35 (Stajkovic et al., 2009). An experimental study shows that team efficacy could influence team-set goal difficulty. That is, teams with higher team efficacy are more likely to set more difficult goals (Durham et al., 1997). Evidence also shows that teams with higher goals tend to perform better (Knight et al., 2001). For the same reason as the individual level, that team goal generation and team goal-striving process are highly correlated, we combine them as a team process. Because it involves the collective regulation of team activities during goal pursuit, we deem it as a team-level parallel concept of individual regulation process and define it as how teams act and react in pursuit of goals. A meta-analysis showed that team processes have strong positive relationships with team performance and that the relationship is similar across different dimensions of team process (i.e., team transition process, team action process, and interpersonal process) (LePine et al., 2008). Thus, a two-step team-level mediating hypothesis is proposed:

**Hypothesis 2**. Team efficacy and team process sequentially mediate the positive influence of group-focused TFL on team performance.

### Cross-Level Effects of Transformational Leadership

While the effects of TFL unfolding with episode are very similar at the individual and team levels, there are cross-level effects. According to open system theory, phenomena of leadership, motivation, and performance at different levels are highly correlated (Chen et al., 2007, 2009; Wang and Howell, 2012). We argue that the interconnection between different-level constructs makes the effects complex. Previous literature suggests that team-level constructs can serve as ambient factors that exert influence on individuals, and team-level variables may have more potent effects in predicting individual-level variables (Chen et al., 2009; Dinh et al., 2014). In addition, a group of scholars argued that higher-level variables might cycle more slowly than lower-level variables (Mathieu and Chen, 2011). Thus, we suggest that the former structures at the team level in the model may have top-down effects on the latter structures at the individual level and eventually affect individual performance.

Several cross-level studies demonstrated that team leadership influences individual performance. For instance, using a service employee sample, Liao and Chuang (2007) found that unit-level TFL is correlated with individual performance, and service climate plays a mediating role. From a social identity perspective, Wang and Howell (2012) suggest that group-focused TFL could promote team-level group identification, and employees who identify with their teams are more likely to perform well. However, little is known about the interplay of different level motivational processes and their effects on individual performance.

First, as stated above, group-focused TFL behaviors aimed at motivating the whole team could boost team efficacy. Hackman (1992) argue that team efficacy performing as ambient stimuli influences how people view their own ability. In teams, no one can evaluate themselves as an independent entity. The interdependent nature of team tasks makes team members' self-efficacy more or less impacted by the team's state. According to the social cognitive theory (Bandura, 2012), one's belief of efficacy can be cultivated through observing efficacious others. It was stated in Chen and Kanfer (2006) that the relationship between team efficacy and self-efficacy is non-recursive. It was argued by Chen et al. (2009) that self-efficacy is generally a more proximal predictor of the individual regulation process due to its greater information value on forming judgments about personal competence. The longitudinal study also demonstrated that some of the variances in individual self-efficacy are explained by group levels of efficacy beliefs about their team (Salanova et al., 2020). Thus, we propose a recursive relationship between team efficacy and self-efficacy and state that group-focused TFL has a positive effect on follower's self-efficacy through the mediating role of team efficacy.

**Hypothesis 3**. Group-focused TFL is positively related to follower's self-efficacy through the mediating role of team efficacy.

Second, due to the interdependent nature of team tasks, one's performance in teams heavily depends on how effectively others played their role. The motivation to accomplish a goal in teams can be influenced by individual motivational states and the team-level motivational states. As argued and demonstrated by Chen et al. (2009) that self-efficacy is a proximal predictor of the individual regulation process due to its greater information value on forming judgments about personal competence, we propose that team efficacy is positively related to the individual regulation process through the mediating role of self-efficacy. In addition, people in teams perceiving a lower team efficacy are less likely to contribute to the team goal. Considering that team tasks are usually highly interdependent, personal goals cannot be entirely separated from the team goal. Individual motivation to accomplish personal goals can be undermined due to the lower motivation to contribute to the team. Thus, we expect that team efficacy is positively related to the individual regulation process through both team process and self-efficacy.

**Hypothesis 4**. Team efficacy is positively related to the individual regulation process through the mediating role of the follower's self-efficacy (H4a) and team process (H4b).

Third, we propose that the team process has cross-level effects on individual performance. For one thing, in teams, individuals must balance their energy on the team task and their own task. An efficient team process featured less team conflict and thus leave more time for team members to accomplish their own goals. However, team inefficiency usually means that team conflict including relational conflict and task conflict exists in the team, which may consume an individual's time and energy to cope with (Costa et al., 2015; Van Knippenberg and Mell, 2016). In addition, according to social cognitive theory, an excellent team process may set norms for individuals to accomplish a specific goal (Hackman, 1992), and hence, work experience gained in teams could promote individual regulation process and individual performance. Thus, we argue that the individual goal-accomplishing process and team performance mediate the relationship between the team goal-accomplishing process and individual performance.

**Hypothesis 5**. The team process is positively related to individual performance through the mediating role of the individual regulation process.

## Methods

### Sample and Procedures

Due to the difficulty of the data collection in teams, a convenient sampling method was used. With the help of the on-the-job graduate students, we got responses from 13 companies that agreed to participate in the survey. The data were collected from permanent work teams of full-time employees from 13 companies in China in diverse industries. The recruiting criteria are that grassroots teams with more than four members (include the leader) and team members have been working together for at least 3 months. Leaders are those who receive reports from their followers and responsible for the team performance. Teams we studied are from the project team, general team, administration team, and others.

We collected the data through web-based surveys and Excel files attached in email conducted four times over 3 months. The team members who could not access the online surveys and all team leaders received the Excel-formatted questionnaires. Data were collected from multiple sources at different times to minimize common method variance. At Time 1, team members completed surveys including dual-level transformational leadership and demographic information. One month later (Time 2), team members rated team efficacy and self-efficacy. Another month later (Time 3), we collected the questionnaires measuring team process and individual regulation process. At Time 4 (1 month later after Time 3), the team leader provided information about the team and member performance, task interdependence, and team type. We matched the multi-stage member responses and their supervisor's ratings by their email addresses from the department of human resource management.

The initial participants included 663 members from 160 teams and their supervisors. Fifty-three, 50, and 48 members did not complete the survey at Time 1, Time 2, and Time 3, respectively. Data from 21 members were dropped due to missing performance rating. Nine teams were omitted because their valid member was only one. Another two teams were omitted due to missing performance rating. The final sample was composed of 477 members from 132 teams and 132 leaders. The valid numbers of respondents per team ranged from 2 to 12 (M = 3.96; SD = 1.80), and the within-team valid response rate ranged from 33 to 100%, with a mean of 87%. Of the 477 members, their mean age was 31.2 (SD = 6.0) years, and 51.2% were women. Their education level was relatively high: 81.9% of members hold a college or higher education degree.

### Measures

#### Dual-Level Transformational Leadership Behavior

Transformational leadership behaviors were assessed by Wang and Howell's (2010) multilevel TFL scale, including the group-focused and individual-focused TFL subscales. The team-level subscale consists of 16 items measuring three dimensions: emphasizing group identity, communicating a group vision, and team-building. A sample item is “(Our direct leader) encourages team members to take pride in our team.” The α for this team-level subscale is 0.96. The individual-level subscale includes 18 items measuring four dimensions: communicating high expectations, follower development, intellectual stimulation, and personal recognition. A sample item is “(My direct leader) challenges me to think about old problems in new ways.” The α for this individual-level subscale is 0.97. All items were rated with a five-point Likert scale ranging from 0 (“not at all”) to 4 (frequently, if not always).

#### Team Efficacy

The team-level efficacy was measured with the four-item collective efficacy scale reported in Salanova et al. (2003). A sample item is “our group is totally competent to solve the task.” Responses were attained on a Likert scale ranging from 1 (“strongly disagree”) to 7 (“strongly agree”). The α for this scale is 0.94.

#### Team Process

This construct was assessed with nine items developed by Mathieu et al. (2006) on a seven-point Likert scale ranging from 1 (“strongly disagree”) to 7 (“strongly agree”). A sample item is “members of our team effectively communicate with each other throughout the workday.” The α for this scale is 0.96.

#### Team Performance

Four items adopted from the five-item team effectiveness scale reported in Tjosvold et al. (2005) were used to assess the team performance by the team leader on a five-point Likert scale ranging from 1 (“strongly disagree”) to 7 (“strongly agree”). A sample item is “team members meet all the formal performance requirements of the job.” The α for this scale is 0.70.

#### Self-Efficacy

Individual-level efficacy was measured with the eight-item general self-efficacy scale developed by Chen et al. (2001). A sample item is “When facing difficult tasks, I am certain that I will accomplish them.” Responses were attained on a Likert scale ranging from 1 (“strongly disagree”) to 7 (“strongly agree”). The α for this scale is 0.95.

#### Individual Regulation Process

Six items were adopted from Chen et al. (2005) to assess individual-level goal generation and striving process on a seven-point Likert scale ranging from 1 (“strongly disagree”) to 7 (“strongly agree”). A sample item is “I identified specific task goals for our team to accomplish.” The α for this scale is 0.93.

#### Individual Performance

Team members' individual performance was assessed by three items from Bono and Judge (2003) by the team leader on a five-point scale ranging from 1 (“needs much improvement”) to 5 (“excellent”). A sample item is “Overall performance in the tasks associated with his/her job (is).”

#### Control Variables

Team members reported demographic variables such as gender, age, education level, and tenure. Team interdependence refers to the degree to which team members must depend on each other to perform their tasks in teams. Teams may vary with regard to the level of the task interdependence between their members. A meta-analysis suggested that task interdependence serves as a moderator of the relationship between leadership and team performance (Burke et al., 2006). Therefore, to test a general motivational model of dual effects of TFL, it is necessary to include task interdependence as a control to illuminate the possible contaminant. The team leader assessed the team task interdependence on two items from Campion et al. (1993) on a five-point scale ranging from 1 (“strongly disagree”) to 5 (“strongly agree”). The diversity of age, gender, and education level for each team member was calculated as additional control variables.

### Aggregation Test

Three team-level variables (group-focused TFL, team efficacy, and team process) fit Chan's (1998) referent shift consensus model where within-group consensus of lower-level elements is required to form higher-level constructs. Thus, we calculated r_wg(j)_ and inter-member reliability (ICC) to justify data aggregation. The results confirmed the aggregation to the team level for all three variables: group-focused TFL behaviors [mean r_wg(j)_ = 0.90; ICC1 = 0.18; ICC2 = 0.44; *F*_(131,345)_ = 1.79, *p* < 0.001], team efficacy [mean r_wg(j)_ = 0.92; ICC1 = 0.08; ICC2 = 0.25; *F*_(131,345)_ = 1.33, *p* < 0.05], and team process [mean r_wg(j)_ = 0.93; ICC1 = 0.20; ICC2 = 0.44; *F*_(131,345)_ = 1.93, *p* < 0.001]. The results also indicated that the ICC (2) values were lower than the traditional 0.70 criterion; the possible reason is the relatively small team size (mean = 3.61) of our valid sample.

### Analytic Strategy

Our data have a multilevel structure with members nested within teams, allowing for both a between-level and a within-level of analysis of covariance. Referring to Liao et al. (2019), we tested the hypothesis by conducting a two-level path analysis within the framework of multilevel structural equation modeling (MSEM; Preacher et al., 2010) using Mplus 7.2 (Muthén and Muthén, 2012), which could partition the variance of the individual-level variables measured into between- and within-team latent components. Firstly, multilevel confirmatory factor analysis (MCFA) was performed to assess the discriminant validity of the employee reported variables at both within and between teams. Then, a two-level path analysis within the framework of multilevel structural equation modeling (MSEM) was used to test our hypotheses.

## Results

### Discriminant Validity

We conducted MCFA of the predictors of individual and team performance with individual-level data for all six variables with parceled factors. A six-factor model, with group-focused TFL, team efficacy, and team process at both the between and the within level and individual-focused TFL, self-efficacy, and individual regulation process at the within level fit the data adequately, ${\text{χ}}_{\left(197\right)}^{2}$ = 356.83, *p* < 0.001; RMSEA = 0.041; CFI = 0.981; TLI = 0.977; SRMR-within = 0.023; SRMR-between = 0.400. All factor loadings were significant at the 0.001 level. Two other models were tested to compare with such a baseline model. Firstly, the two-factor model with three team-level variables loading onto one factor at the between and the within level and three individual-level variables loading onto another factor at the within level provided poorer fit than our baseline model [Satorra-Bentler Scaled ${\text{χ}}_{\left(17\right)}^{2}$ = 1795.36, *p* < 0.001], ${\text{χ}}_{\left(214\right)}^{2}$ = 5298.15, *p* < 0.001; RMSEA = 0.223; CFI = 0.386; TLI = 0.326; SRMR-within = 0.294; SRMR-between = 0.865. Then, in the third model, we loaded group-focused TFL and individual-focused TFL onto one TFL factor and kept the other factors consistent with the baseline model. This third model also exhibited a poorer fit than our baseline model [Satorra-Bentler Scaled ${\text{χ}}_{\left(51\right)}^{2}$ = 320.56, *p* < 0.001].

### Hypotheses Testing

Table 1 presents the means, standard deviations, reliability coefficients, and correlations of the variables at the individual and team levels. At the team level, of the correlations between team performance and control variables, only the one between task interdependence and team performance is significant. Then, task interdependence was included in the MSEM as the control variable.

**Table 1:** Descriptive statistics and correlations.

**Variables**	***M***	***SD***	**1**	**2**	**3**	**4**	**5**	**6**	**7**	**8**	**9**
**Individual-level variables (*****N*** **=** **477)**										
1. Gender	0.49	0.50									
2. Age	31.19	5.99	−0.01								
3. Education	0.91	0.52	0.13^**^	−0.14^**^							
4. Individual-focused TFL (T1)	2.52	0.79	0.04	−0.24^**^	0.04	**0.96**					
5. Self-efficacy (T2)	5.72	0.79	−0.01	−0.06	−0.01	0.28^**^	**0.95**				
6. Individual regulation process (T3)	5.70	0.74	−0.02	0.02	−0.01	0.34^**^	0.46^**^	**0.93**			
7. Individual performance (T4)	3.60	0.93	0.09	0.08	−0.06	0.14^**^	0.15^**^	0.34^**^			
**Team-level variables (*****N*** **=** **132)**										
1. Team size	3.61	1.76									
2. Task interdependence	4.33	0.65	−0.17^*^	**0.76**							
3. Age diversity	0.13	0.07	−0.05	0.03							
4. Gender diversity	0.28	0.22	0.05	−0.03	0.16						
5. Education diversity	0.25	0.23	0.02	0.05	0.11	0.15					
6. Group-focused TFL (T1)	2.60	0.49	0.04	0.24^**^	0.00	0.06	−0.01	**0.96**			
7. Team-efficacy (T2)	5.83	0.49	0.04	−0.03	−0.08	0.11	−0.03	0.36^**^	**0.94**		
8. Team process (T3)	5.64	0.57	0.02	0.17	0.04	−0.02	0.05	0.40^**^	0.49^**^	**0.96**	
9. Team performance (T4)	4.10	0.46	0.05	0.32^**^	0.07	−0.04	0.06	0.09	0.37^**^	0.36^**^	**0.70**

*Internal consistency reliabilities appear along the diagonal in bold*.

* *p < 0.05*.

** *p < 0.01*.

**Figure 1:**
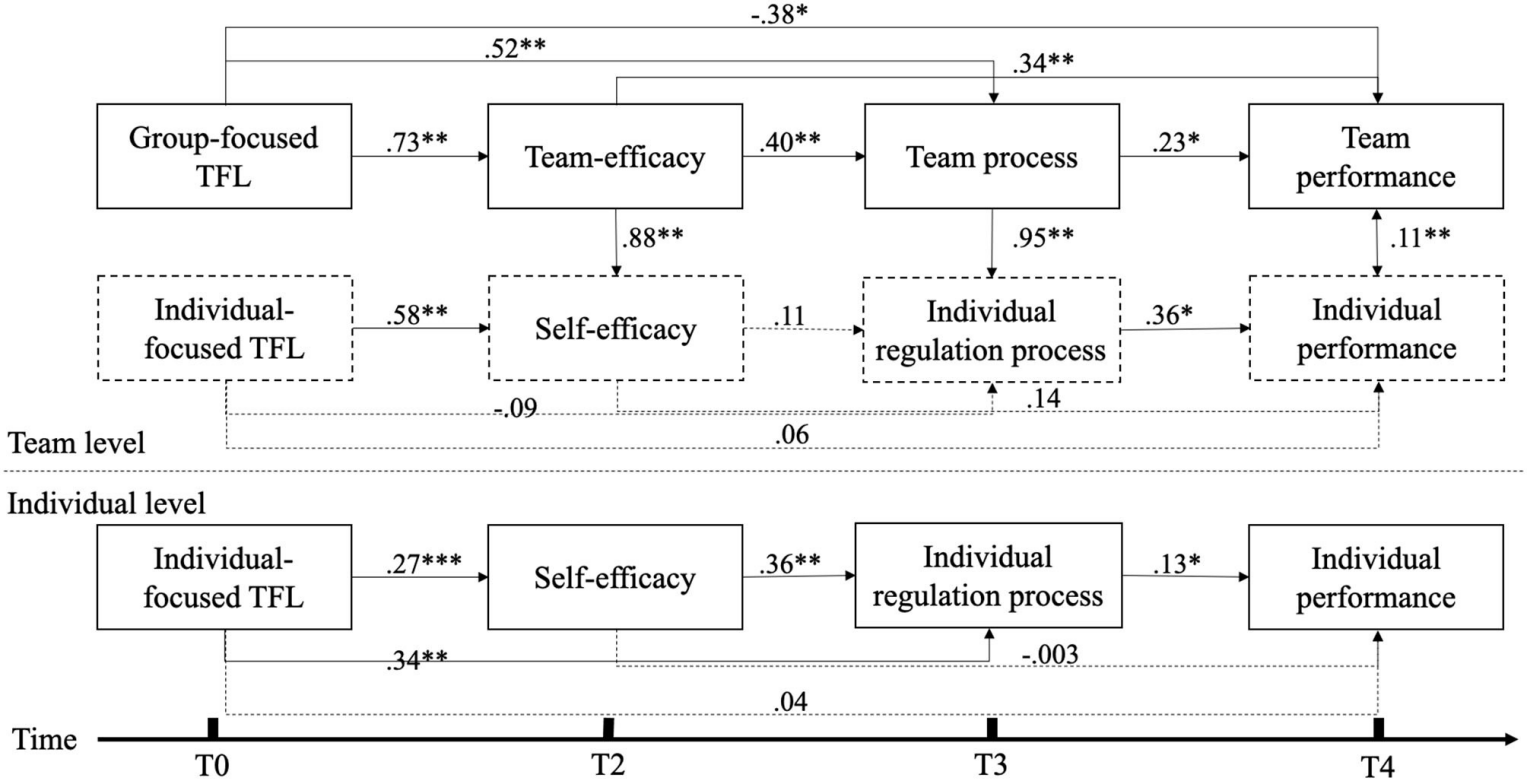
Results of multilevel SEM without latent constructs. Standardized path coefficients are presented. *p < 0.05; **p < 0.01; ***p < 0.001.

To test our hypotheses, we conducted MSEM in Mplus with aggregated team-level variables (group-focused TFL, team efficacy, and team process) in the between-level and individual-level variables (individual-focused TFL, self-efficacy, and individual regulation process) in the between and the within level, as shown in Figure 1. This structural model fit the sample data well, ${\text{χ}}_{\left(25\right)}^{2}$ = 79.12, *p* < 0.001; RMSEA = 0.067; CFI = 0.924; TLI = 0.845; SRMR-within = 0.018; SRMR-between = 0.052. Tables 2, 3 present the unstandardized estimates of direct and indirect effects, respectively. Figure 1 shows the results of the full structural model with the standardized path coefficients.

**Table 2:** Unstandardized estimates of direct effects.

	**Self-efficacy**		**Individual regulation process**		**Individual performance**		**Team efficacy**		**Team process**		**Team performance**	
	**Estimates**	***p***	**Estimates**	***p***	**Estimates**	***p***	**Estimates**	***p***	**Estimates**	***p***	**Estimates**	***p***
**Individual level**												
Gender					0.02	0.695						
Age					0.01	0.046						
Education					0.00	0.969						
Individual-focused TFL	0.18	<0.001	0.21	<0.001	0.03	0.583						
Self-efficacy			0.32	<0.001	−0.01	0.836						
Individual regulation process					0.12	0.027						
**Team level**												
Individual-focused TFL	0.21	0.002	−0.04	0.538	0.04	0.784						
Self-efficacy			0.13	0.107	0.23	0.505						
Individual regulation process					0.66	0.011						
Task interdependence											0.19	<0.001
Group-focused TFL							0.36	<0.001	0.29	0.005	−0.17	0.039
Team efficacy	0.66	<0.001							0.46	<0.001	0.31	<0.001
Team process			0.71	<0.001							0.18	0.020

**Table 3:** Unstandardized estimates of indirect effects.

**Paths**	**Estimates**	***p* value**	**LLCI**	**ULCI**
**Individual level**				
Individual-focused TFL → Self-efficacy → Individual regulation process → Individual performance (H1)	0.007	0.035	0.001	0.014
Individual-focused TFL → Self-efficacy → Individual performance	−0.002	0.836	−0.017	0.014
Individual-focused TFL → Individual regulation process → Individual performance	0.025	0.041	0.001	0.048
**Team level**				
Individual-focused TFL → Self-efficacy → Individual regulation process → Individual performance (H1)	0.018	0.224	−0.011	0.047
Individual-focused TFL → Self-efficacy → Individual performance	0.049	0.526	−0.102	0.199
Individual-focused TFL → Individual regulation process → Individual performance	−0.025	0.556	−0.110	0.059
Group-focused TFL → Team efficacy → Team process → Team performance (H2)	0.030	0.033	0.002	0.057
Group-focused TFL → Team efficacy → Team performance	0.112	0.005	0.033	0.191
Group-focused TFL → Team process → Team performance	0.054	0.090	−0.008	0.116
**Cross-level**				
Group-focused TFL → Team efficacy → Self-efficacy (H3)	0.238	<0.001	0.117	0.359
Team-efficacy → Self-efficacy → Individual regulation process (H4a)	0.084	0.114	−0.020	0.189
Team efficacy → Team process → Individual regulation process (H4b)	0.325	<0.001	0.184	0.466
Team process → Individual regulation process → Individual performance (H5)	0.472	0.005	0.139	0.806

Hypothesis 1 predicted that individual-focused TFL has a positive effect through self-efficacy and the individual regulation process. This is a 1-1-1-1 mediation hypothesis. As shown in Table 2, at the individual level, individual-focused TFL is positively related to self-efficacy (γ = 0.18, *p* < 0.001), which is positively correlated with individual regulation process (γ = 0.32, *p* < 0.001). Also, there is a positive correlation between the individual regulation process and individual performance (γ = 0.12, *p* < 0.05). Moreover, as shown in Table 3, there is a significant indirect effect from individual-focused TFL to individual performance (Individual-focused TFL → Self-efficacy → Individual regulation process → Individual performance) indicated by the Monte Carlo confidence intervals (CIs; [0.001, 0.014]) not containing a zero, thus supporting Hypothesis 1 at the individual level. However, at the team level, the correlation between self-efficacy and individual regulation process is not significant (γ = 0.13, *p* = 0.107), and the CIs [−0.011, 0.047] of this indirect effect contained zero, thus not supporting Hypothesis 1.

Hypothesis 2 stated that group-focused TFL had a positive effect on team performance through team efficacy and team process serially. The results in Table 2 show that group-focused TFL correlates positively with team efficacy (γ = 0.36, *p* < 0.001), which positively relates to team process (γ = 0.46 *p* < 0.001). Also, the team process has a positive effect on team performance (γ = 0.18, *p* < 0.05). In addition, as shown in Table 3, there is a significant indirect effect from group-focused TFL to team performance (Group-focused TFL → Team efficacy → Team process → Team performance) indicated by the CIs [0.002, 0.057] not containing a zero, thus supporting Hypothesis 2.

In the cross-level, as shown in Table 2, team efficacy is positively related to self-efficacy (γ = 0.66, *p* < 0.001), and team process positively correlated with the individual regulation process (γ = 0.71, *p* < 0.001). These significant correlations lay a solid foundation for the cross-level mediation analysis. The results of bootstrap analysis shown in Table 3 provides further evidence for H3, H4b, and H5 with the CIs not containing a zero. Specifically, group-focused TFL has a positive effect on self-efficacy through team efficacy (CIs [0.117, 0.359]; H3); team efficacy shows a positive effect on the individual regulation process through team process (CIs [0.184,0.466]; H4b); and team process has a positive effect on individual performance through the individual regulation process (CIs [0.139,0.806]; H5). The mediation effect of self-efficacy between team efficacy and individual regulation process (H4a) is not supported due to the non-significant correlation between individual regulation process and self-efficacy (γ = 0.13, *p* < 0.10) at the team level.

### Supplementary Analysis

Referring to Spector and Brannick (2011), we also conducted a MSEM analysis without any control variables to test the sensitivity of the present model. The structural model fit the sample data well, ${\text{χ}}_{\left(12\right)}^{2}$ = 10.743, *p* = 0.551; RMSEA = 0.000; CFI = 1.000; TLI = 1.005; SRMR-within = 0.008; SRMR-between = 0.003. Tables 4, 5 present the unstandardized estimates of direct and indirect effects of this model, respectively.

**Table 4:** Unstandardized estimates of direct effects without control variables.

	**Self-efficacy**		**Individual regulation process**		**Individual performance**		**Team efficacy**		**Team process**		**Team performance**	
	**Estimates**	***p***	**Estimates**	***p***	**Estimates**	***p***	**Estimates**	***p***	**Estimates**	***p***	**Estimates**	***p***
**Individual level**												
Individual-focused TFL	0.19	<0.001	0.21	<0.001	0.01	0.830						
Self-efficacy			0.32	<0.001	−0.01	0.820						
Individual regulation process					0.12	0.022						
**Team level**												
Individual-focused TFL	0.21	0.001	−0.03	0.634	0.03	0.834						
Self-efficacy			0.13	0.109	0.52	0.606						
Individual regulation process					0.70	0.005						
Group-focused TFL							0.36	<0.001	0.30	0.005	−0.11	0.178
Team efficacy	0.66	<0.001							0.46	<0.001	0.26	0.007
Team process			0.71	<0.001							0.22	0.008

**Table 5:** Unstandardized estimates of indirect effects without control variables.

**Paths**	**Estimates**	***p* value**	**LLCI**	**ULCI**
**Individual level**				
Individual-focused TFL → Self-efficacy → Individual regulation process → Individual performance (H1)	0.007	0.032	0.001	0.014
Individual-focused TFL → Self-efficacy → Individual performance	−0.002	0.820	−0.018	0.014
Individual-focused TFL → Individual regulation process → Individual performance	0.026	0.036	0.002	0.049
**Team level**				
Individual-focused TFL → Self-efficacy → Individual regulation process → Individual performance (H1)	0.019	0.209	−0.010	0.048
Individual-focused TFL → Self-efficacy → Individual performance	0.038	0.617	−0.109	0.184
Individual-focused TFL → Individual regulation process → Individual performance	−0.020	0.643	−0.105	0.065
Group-focused TFL → Team efficacy → Team process → Team performance (H2)	0.036	0.019	0.006	0.066
Group-focused TFL → Team efficacy → Team performance	0.094	0.025	0.012	0.177
Group-focused TFL → Team process → Team performance	0.065	0.071	−0.006	0.135
**Cross-level**				
Group-focused TFL → Team efficacy → Self-efficacy (H3)	0.238	<0.001	0.117	0.359
Team-efficacy → Self-efficacy → Individual regulation process (H4a)	0.083	0.116	−0.021	0.188
Team efficacy → Team process → Individual regulation process (H4b)	0.325	<0.001	0.185	0.466
Team process → Individual regulation process → Individual performance (H5)	0.494	0.002	0.175	0.814

As shown in Table 4, at the individual level, individual-focused TFL is positively related to self-efficacy (γ = 0.19, *p* < 0.001), which is positively correlated with individual regulation process (γ = 0.32, *p* < 0.001). Also, there is a positive correlation between the individual regulation process and individual performance (γ = 0.12, *p* < 0.05). Moreover, as shown in Table 3, there is a significant indirect effect from individual-focused TFL to individual performance (Individual-focused TFL → Self-efficacy → Individual regulation process → Individual performance) indicated by the Monte Carlo confidence intervals (CIs; [0.001, 0.014]) not containing a zero, thus supporting Hypothesis 1 at the individual level. However, at the team level, the correlation between self-efficacy and individual regulation process is not significant (γ = 0.13, *p* = 0.109) and the CIs [−0.010, 0.048] of this indirect effect contained zero, thus not supporting Hypothesis 1.

The results in Table 4 show that group-focused TFL correlates positively with team efficacy (γ = 0.36, *p* < 0.001), which positively relates to team process (γ = 0.46 *p* < 0.001). Also, the team process has a positive effect on team performance (γ = 0.22, *p* < 0.05). In addition, as shown in Table 3, there is a significant indirect effect from group-focused TFL to team performance (Group-focused TFLTeam efficacyTeam processTeam performance) indicated by the CIs [0.006, 0.066] not containing a zero, thus supporting Hypothesis 2.

In the cross-level, as shown in Table 2, team efficacy is positively related to self-efficacy (γ = 0.66, *p* < 0.001), and team process positively correlated with the individual regulation process (γ = 0.71, *p* < 0.001). These significant correlations lay a solid foundation for the cross-level mediation analysis. The results of bootstrap analysis shown in Table 3 provide further evidence for H3, H4b, and H5 with the CIs not containing a zero. Specifically, group-focused TFL has a positive effect on self-efficacy through team efficacy (CIs [0.117, 0.359]; H3); team efficacy shows a positive effect on the individual regulation process through team process (CIs [0.185, 0.466]; H4b); and team process has a positive effect on individual performance through the individual regulation process (CIs [0.175, 0.814]; H5). The mediation effect of self-efficacy between team efficacy and individual regulation process (H4a) is not supported due to the non-significant correlation between individual regulation process and self-efficacy (γ = 0.13, *p* = 0.109) at team level.

## Discussion

The aim of the present study is to explore the motivational effects of TFL on followers and determine how structures in different levels interplay in this process. By integrating dual-level TFL and a multilevel model of motivation in teams, a multilevel model of motivational effects of TFL is proposed and tested in this study. The results supported most of our proposals. At the individual level, individual-focused TFL has positive effects on followers' self-efficacy, which promotes the individual regulation process and results in high-level individual performance. Similarly, at the team level, team-focused transformational leadership could enhance team efficacy. Teams with high team efficacy will do better in team process and result in high-level team performance. In addition, we found cross-level effects among structures at different levels. Group-focused TFL positively influences follower self-efficacy through the mediating role of team efficacy, team efficacy positively influences individual regulation process through its influence on team process, and team process could positively influence individual performance through promoting individual regulation process.

### Theoretical Implications

The results of the present study have important implications for research in leadership, motivation, and teamwork. First, the current study provides empirical evidence for the dual effects of TFL beyond the previous literature. As leadership is inherently multilevel (Yammarino and Dansereau, 2008), it affects both individuals and the team as a whole. This study examining leadership focusing at both individual and team level contributes beyond previous literature. For example, in Wu et al. (2010), leadership is examined solely at the team level and revealed that leaders exhibiting varying levels of individual-focused behavior might diminish group effectiveness through creating divergence in employee's identification and self-efficacy, while group-focused leadership can promote group effectiveness by facilitating member's group identification and team efficacy. However, the present study tested dual effects of TFL from a different angle by examining parallel motivational effects of team-focused TFL and individual-focused TFL. Therefore, this study responded to the continuing calls for examining the multilevel effects of leadership (Dinh et al., 2014). Also, the present study contributes beyond Wu et al. (2010) by demonstrating that individual-focused TFL has positive effects on the self-efficacy and self-regulation process, which results in positive individual outcomes. Besides, consistent with Wang and Zhu (2011) and Wang and Howell (2010, 2012), the results suggested that group-focused TFL positively affects team performance through the team motivation process, and individual-focused transformational leadership positively affects individual performance through its influence on the individual motivation process. The present study contributes beyond Wang and Howell (2012) by demonstrating the two-step mediation of motivational state and team (individual) process in the relationship between dual-level transformational leadership and team (individual) performance. Moreover, it is also indicated in our study that group-focused TFL has critical “spillover” effects on individual-level motivation and outcomes. Group-focused TFL indirectly affects followers' self-efficacy through team efficacy. When a leader displays group-focused TFL, the whole team will have more confidence in the ability of their team to handle a specific task. The interdependent nature of tasks in teams makes the accomplishment of a personal job depend on others (Bertucci et al., 2016). So, due to the confidence in their teams, individuals will be more confident in themselves in doing their jobs in teams.

Second, the results of our study provide empirical evidence for the multilevel model of motivation in teams proposed by Chen and Kanfer (2006). The present study, which considers the influence of contextual factors on individual and team motivation, as well as motivation outcomes, revealed a detailed pathway through which TFL works overtime at both levels. TFL behavior measured at Time 1 is positively related to efficacy measured at Time 2, which promotes the process (measured at Time 3) and performance (measured at Time 4) at both individual and team levels. As suggested by Marks et al. (2001) and Chen and Kanfer (2006), examining motivational processes as they unfold over time could enrich our understanding of behaviors in complex social systems. By examining the cross-level effects of motivational structure, our results provide support for the time-lagged top-down effects of motivation. Group-focused TFL at the former stage positively affects the latter stage individual self-efficacy through the mediating role of team efficacy. Team efficacy at the former stage positively influences the individual regulation process at the latter stage through its effects on the team process. The team process at the former stage positively affects individual performance at the latter stage through its influence on individual performance.

Third, combining dual-level transformational leadership with a multilevel motivation model, the present study gives more detail to teamwork models such as the traditional Input-Process-Outcome (IPO) model and later Input-Mediator-Outcome (IMO) model devised based on the IPO model (Ilgen et al., 2005; Mathieu et al., 2008). The IMO model of team effectiveness suggests that mediators of team effectiveness involve emergent state and team process. In our study, taking dual-level TFL as an input, motivational states and individual or team processes as mediators, and individual and team performance as output, we confirmed the IMO model. Furthermore, the multilevel and cross-level effects we found enriched the IMO model. As individuals are important components of teams, both individual and team functions should be considered simultaneously to improve team effectiveness. The cross-level effects of team-level structures on individual-level structures found in this study advance the teamwork model, which is not linear but reticulates and includes processes that unfold over episodes and across levels.

### Managerial Implications

There are some important practical implications of our study. Since organizations highlight both team performance and individual performance, leaders have responsibilities to motivate both of these two aspects. However, leaders usually have limited resources to pay attention to both and must compromise in some circumstances. In this study, we suggest that transformational leadership focused on different levels uniquely and jointly influences follower performance. Individual-focused TFL could promote individual performance, whereas group-focused TFL could promote team performance and has “spillover” effects on individual-level motivation and outcomes. This evidence indicates that, when leaders must compromise between energy invested in the team and individual followers, group-focused TFL is a more efficient way to maximize benefits (Wang and Howell, 2012).

Work motivations in the middle of leadership and followers' performance play a key role in enhancing team effectiveness and individual performance. It suggests that organizations and managers should make efforts to promote followers' motivation to make them commit to their work and coordinate to contribute to the team. A key part of work motivation is efficacy. To promote individual performance, managers should pay more attention to building followers' confidence by developing followers' knowledge and skills or intellectual stimulation. To promote team performance, managers should try to build team efficacy (Stajkovic et al., 2009). However, as the moderate level of team efficacy is likely to have the greatest effect on performance (Park et al., 2017), practitioners should consider the extent of followers' and teams' efficacy.

As our data were collected in the Chinese context, this study has special implications for Chinese companies. Results of this study provide direct evidence for Chinese companies adopting a dual-level TFL, which could enhance team performance and individual performance as a whole. There is an entrenched tendency of leaders in China to address collective interests while overlooking the individual needs of their followers. In particular, new-generation employees ask for more self-recognition in the workplace (Warner and Zhu, 2018). Addressing team value exclusively is not wise. This study demonstrates that leadership behavior focused on the individual level also matters, as it could motivate individual followers to attain their own goals and enhance individual performance. Thus, it is beneficial for team leaders in the Chinese context adopting a dual-level TFL.

### Limitations and Future Research

Some limitations of this study should be noted. First, several effects proposed in our model may be exaggerated. For example, the cross-level effects we found in the present study may be exaggerated because of the inevitable common method bias. Structures at the individual level are collected at the same time as their team-level counterparts, and the relatively high correlations between these structures in the results foreshadow this tendency. Further study to solidify this model should try to avoid this drawback through methods such as having half of the team members rate variables at the team level and another half rate the individual-level variables. In addition, the effects of TFL on performance may be exaggerated because it is possible that prior performance may influence the final performance. Evidence suggests that prior performance positively influences subsequence individual performance (Chen et al., 2009). Thus, to attain solid conclusions, future research should be conducted with a well-controlled prior performance of both levels.

Second, dual-level TFL *per se* has limitations because it distinguishes individual- and group-focused TFL behavior but does not consider behaviors that affect both levels. Scholars suggested that behaviors to promote performance at individual and team levels may have some overlap (Schriesheim et al., 2009). For example, intellectual stimulation, defined as a dimension of individual-focused TFL, is proposed to affect both individual and group levels. Future studies are expected to extend the current model of dual-level transformational by considering items that affect both levels.

Third, we did not explore the possible boundary conditions for the effects of dual-level TFL. Further study should explore possible moderators to specify this model. For example, task interdependence as a critical moderator of team leadership effects has been tested in many studies. Li et al. (2016) found that the effects of dual-level TFL on individual and team innovation are moderated by task interdependence. Specifically, the negative effects of group-focused TFL on individual innovation will be enhanced when there is high task interdependence in the team. It is possible that conditional factors like task interdependence may serve as a moderator in the current model, as it may influence the way members in a team coordinate with each other. Also, the relationship between leaders and subordinates may influence the effectiveness of leadership. Factors like leader–member exchange and leader–team exchange are also supposed to influence the way dual-level TFL takes effect. For example, it has been demonstrated that in a higher LMX differentiation condition, teams with a high mean leader–member relationship still have some problem in team conflict in contrast with lower LMX differentiation condition (Boies and Howell, 2006). Future studies should go further to test possible conditional factors beyond the current model.

Fourth, we did not address other antecedents or mechanisms that may influence follower performance, such as cognitive mechanisms [e.g., knowledge sharing, see Dong et al. (2017)] and behavior mechanisms [e.g., helping behavior, see Lorinkova and Perry (2018)]. Fully considering these mechanisms could help demonstrate the possible incremental validity of the motivational mechanism we proposed. In addition, self-efficacy is only one part of motivational states. Other structures, such as psychological empowerment, potency, and cohesion (Marks et al., 2001; Chen and Kanfer, 2006), were not considered in the present study. Further study may contribute by addressing these factors in the present model.

Fifth, in our study, only the top-down effects of TFL were examined. However, research suggested that differentiated TFL is detrimental to team performance (Wu et al., 2010). A study of top manager teams revealed that differentiated leadership could undermine both team effectiveness and firm performance (Zhang et al., 2015) because CEO's different treatment of TMT members increases the discrepancy among them and disrupt the team's dynamics, which results in lower follower-rated team effectiveness and firm performance. Thus, further study is expected to examine the possible bottom-up effects of individual-focused TFL (Dinh et al., 2014).

Finally, although variables were measured at four separate time points in a sequence to match the theoretical causal hypotheses, causal conclusions should be made with great caution. Future studies may adopt a longitudinal design to reach a solid causal conclusion.

## Data Availability Statement

The raw data supporting the conclusions of this article will be made available by the authors, without undue reservation.

## Ethics Statement

The studies involving human participants were reviewed and approved by Ethics Committee of the Institute of Psychology, Chinese Academy of Sciences. The patients/participants provided their online informed consent to participate in this study.

## Author Contributions

HL contributed to the conceptualization of study, interpretation of results, and drafted and made substantive edits and revisions to the manuscript. FL guided the conceptualization of the study design, collected and analyzed the data, and contributed to manuscript preparation and revision. Both authors contributed to the article and approved the submitted version.

## Conflict of Interest

The authors declare that the research was conducted in the absence of any commercial or financial relationships that could be construed as a potential conflict of interest.
